# Transcriptomic Features Predict Survival in Glioblastoma Patients

**DOI:** 10.7759/cureus.113401

**Published:** 2026-07-26

**Authors:** Michelle A Nakatsuka, Frank Liu

**Affiliations:** 1 Neurological Surgery, New York University Grossman School of Medicine, New York, USA; 2 Oncology, New York University Grossman School of Medicine, New York, USA

**Keywords:** bioinformatics, cox regression, gene expression, glioblastoma, kaplan-meier analysis, lasso regression, prognostic biomarkers, survival prediction, tcga, transcriptomics

## Abstract

Background: Glioblastoma (GBM) is the most aggressive primary malignant brain tumor in adults and demonstrates substantial transcriptomic heterogeneity associated with patient survival. High-dimensional genomic datasets present statistical challenges because the number of measured molecular features often exceeds the number of available patient samples. Penalized regression methods such as Least Absolute Shrinkage and Selection Operator (LASSO) regression enable simultaneous feature selection and survival modeling in these settings.

Methods: Transcriptomic expression and survival data from The Cancer Genome Atlas (TCGA) GBM cohort were analyzed using LASSO-regularized Cox proportional hazards regression implemented through the glmnet package in R. After preprocessing and sample matching, 518 tumor samples and 12,042 transcriptomic features were retained for analysis. Patients were randomly divided into training (n=414) and test (n=104) cohorts. Ten-fold cross-validation using Harrell’s concordance index (C-index) was performed to identify optimal regularization parameters. Kaplan-Meier survival analysis was used to evaluate risk stratification performance, and receiver operating characteristic (ROC) analysis was performed for selected candidate genes.

Results: Cross-validation identified a maximum C-index of 0.583 at the optimal lambda.min regularization parameter. The final LASSO-Cox model retained 74 genes with nonzero coefficients. Two identified genes, CLEC5A and RANBP17, overlapped with previously reported GBM prognostic genes from an independent study. Kaplan-Meier analysis demonstrated significant survival separation between predicted high-risk and low-risk groups in both the training cohort (log-rank p < 0.0001) and the held-out test cohort (log-rank p < 0.05). ROC analysis of selected candidate genes demonstrated moderate discriminatory performance, with area under the curve values ranging from 0.604 to 0.701.

Conclusions: LASSO-regularized Cox regression identified sparse transcriptomic features associated with survival in TCGA GBM patients. Survival stratification performance persisted in a held-out test dataset, supporting the reproducibility of the derived transcriptomic risk model. These findings demonstrate the applicability of penalized survival modeling approaches to high-dimensional cancer transcriptomic datasets and support further investigation of transcriptomic biomarkers in GBM prognosis.

## Introduction

Glioblastoma (GBM) is the most common and aggressive primary malignant brain tumor in adults and is thought to arise from astrocytic lineage cells. It is characterized by highly infiltrative growth, making complete surgical resection nearly impossible and contributing to frequent recurrence despite multimodal treatment consisting of maximal safe surgical resection, radiation therapy, and chemotherapy. Consequently, overall survival (OS) remains poor despite current standard-of-care therapy. Established prognostic factors such as age, performance status, MGMT promoter methylation, and IDH mutation status provide important clinical information but incompletely capture the marked biological heterogeneity observed across GBM tumors. These limitations have motivated continued investigation of molecular biomarkers capable of improving prognostic stratification [1,2]. Median survival remains approximately 12-15 months even with current standard-of-care therapy, highlighting the need for improved prognostic stratification and therapeutic targeting [3]. GBM demonstrates substantial molecular and clinical heterogeneity, with considerable variation in patient outcomes despite similar histopathologic diagnoses [4]. As a result, increasing attention has been directed toward the use of transcriptomic (genome-wide gene expression) and genomic profiling approaches to identify biomarkers associated with prognosis, including immune-related, extracellular matrix-related, and metabolic gene signatures reported in prior studies [5,6].

Large-scale cancer genomics initiatives such as The Cancer Genome Atlas (TCGA) have enabled the generation of high-dimensional transcriptomic datasets containing expression measurements for thousands of genes across GBM cohorts [7]. Although these datasets provide opportunities for biomarker discovery and prognostic modeling, they also introduce substantial statistical challenges because the number of measured genomic features frequently exceeds the number of patient samples. Traditional Cox proportional hazards regression models may overfit under these high-dimensional conditions and may produce unstable coefficient estimates when applied to transcriptomic data [8].

Penalized regression approaches such as Least Absolute Shrinkage and Selection Operator (LASSO) regression have emerged as important tools for high-dimensional survival modeling because they simultaneously perform coefficient shrinkage and feature selection [9]. By constraining less informative coefficients toward zero, LASSO-based Cox proportional hazards models can reduce overfitting while identifying sparse sets of prognostically relevant genes [10]. Prior studies have applied LASSO-based transcriptomic modeling approaches to GBM and other malignancies to identify survival-associated molecular signatures and improve prognostic stratification [11-13]. However, many previously proposed prognostic signatures demonstrate limited interpretability, inconsistent reproducibility across datasets, or inadequate validation in independent cohorts.

In this study, LASSO-regularized Cox proportional hazards regression was applied to TCGA GBM transcriptomic data to identify sparse gene-expression signatures associated with OS. Cross-validation was used to determine optimal regularization parameters, and receiver operating characteristic (ROC) analysis was performed for selected genes associated with survival classification. By combining penalized survival modeling with transcriptomic feature selection, this study aimed to identify prognostically relevant molecular features while reducing dimensionality and improving model interpretability.

## Materials and methods

Gene expression, copy number variation (CNV), and clinical survival data were obtained from The Cancer Genome Atlas Glioblastoma Multiforme cohort (TCGA-GBM) through the UCSC Xena Browser (University of California Santa Cruz, Santa Cruz, CA, USA) on April 30, 2026 [14,15]. Transcriptomic expression data were downloaded from the TCGA.GBM.sampleMap/HT_HG-U133A dataset [16], while CNV data were obtained from the TCGA.GBM.sampleMap/Gistic2_CopyNumber_Gistic2_all_data_by_genes dataset [17]. Clinical survival information was obtained from the survival_GBM_survival dataset. OS was used as the primary survival endpoint. Survival time was measured in days from diagnosis to death or last follow-up, consistent with TCGA clinical survival annotations. The HG-U133A microarray cohort was selected because of its larger number of samples with matched survival data compared with the RNA-sequencing cohort, thereby improving statistical power for downstream survival modeling. Samples lacking OS information or with OS times less than or equal to zero days were excluded. Gene expression and survival datasets were harmonized using shared TCGA sample identifiers, and only patients with both transcriptomic and survival data available were retained for analysis. After filtering and harmonization, 518 patients were included in the final cohort.

Transcriptomic preprocessing consisted of matrix alignment and filtering to ensure consistency across expression and survival datasets. No additional normalization beyond the processed UCSC Xena expression matrices was performed because the downloaded dataset consisted of provider-preprocessed, log2-transformed microarray expression values. OS was used as the primary survival endpoint. Survival time (OS time) was measured in days, and survival status (OS) was coded according to TCGA survival annotations.

LASSO-regularized Cox proportional hazards regression was performed to identify transcriptomic features associated with GBM survival. Statistical analyses were performed in R version 4.5.1 (R Foundation for Statistical Computing, Vienna, Austria) using RStudio version 2025.05.1+513 (Posit Software, Boston, MA, USA) using the glmnet and survival packages [18-21].

Patients were randomly divided into training (80%) and held-out testing (20%) cohorts using a fixed random seed (seed = [1]) for reproducibility. The training cohort consisted of 414 patients, while the testing cohort consisted of 104 patients. Model training was performed exclusively on the training cohort.

A Cox proportional hazards model with LASSO regularization was fit using the glmnet package. Ten-fold cross-validation was performed using the cv.glmnet function with concordance index (C-index) optimization (type.measure = "C") to determine the optimal regularization parameter (λ). Both the minimum cross-validation error model (λ_min) and the more parsimonious one-standard-error model (λ_1se) were evaluated to assess the tradeoff between predictive performance and model sparsity. Additional mathematical details regarding the LASSO-regularized Cox proportional hazards framework and penalized likelihood optimization are provided in the Appendix Methods.

Genes with nonzero coefficients in the optimized LASSO-Cox model were identified as candidate prognostic transcriptomic features. Coefficient path plots and cross-validated performance curves were generated to visualize feature selection behavior across regularization strengths.

Model performance was evaluated on the held-out test cohort using concordance index analysis. Predicted risk scores were generated from the optimized LASSO-Cox model and used to stratify patients into high-risk and low-risk groups according to the median predicted risk score.

Kaplan-Meier survival analyses were performed using the survival and survminer packages in R [21,22]. Differences between survival curves were assessed using the log-rank test. Kaplan-Meier plots were generated for both training and testing cohorts to evaluate survival separation between predicted risk groups.

ROC analysis was performed for selected high-impact prognostic genes identified by the LASSO-Cox model. Patients were categorized according to 2.5-year survival status. Samples censored prior to the cutoff time were excluded from ROC analysis.

Logistic regression was used solely to evaluate discrimination at the predefined 2.5-year survival endpoint. Although time-dependent ROC analysis represents an alternative approach for survival prediction, the fixed-time approach was selected to provide a straightforward assessment of clinically relevant survival classification and facilitate comparison of candidate transcriptomic features. Logistic regression models were constructed individually for selected genes, and ROC curves were generated using the pROC package in R [23]. Area under the curve (AUC) values were calculated to assess discriminatory performance for survival classification.

All statistical analyses and figure generation were performed in R using the glmnet, survival, survminer, ggplot2, dplyr, and pROC packages [18-23]. Cross-validation plots, Kaplan-Meier survival curves, coefficient path plots, null deviance plots, and ROC curves were generated programmatically within the analysis pipeline to ensure reproducibility.

## Results

**Figure 1:**
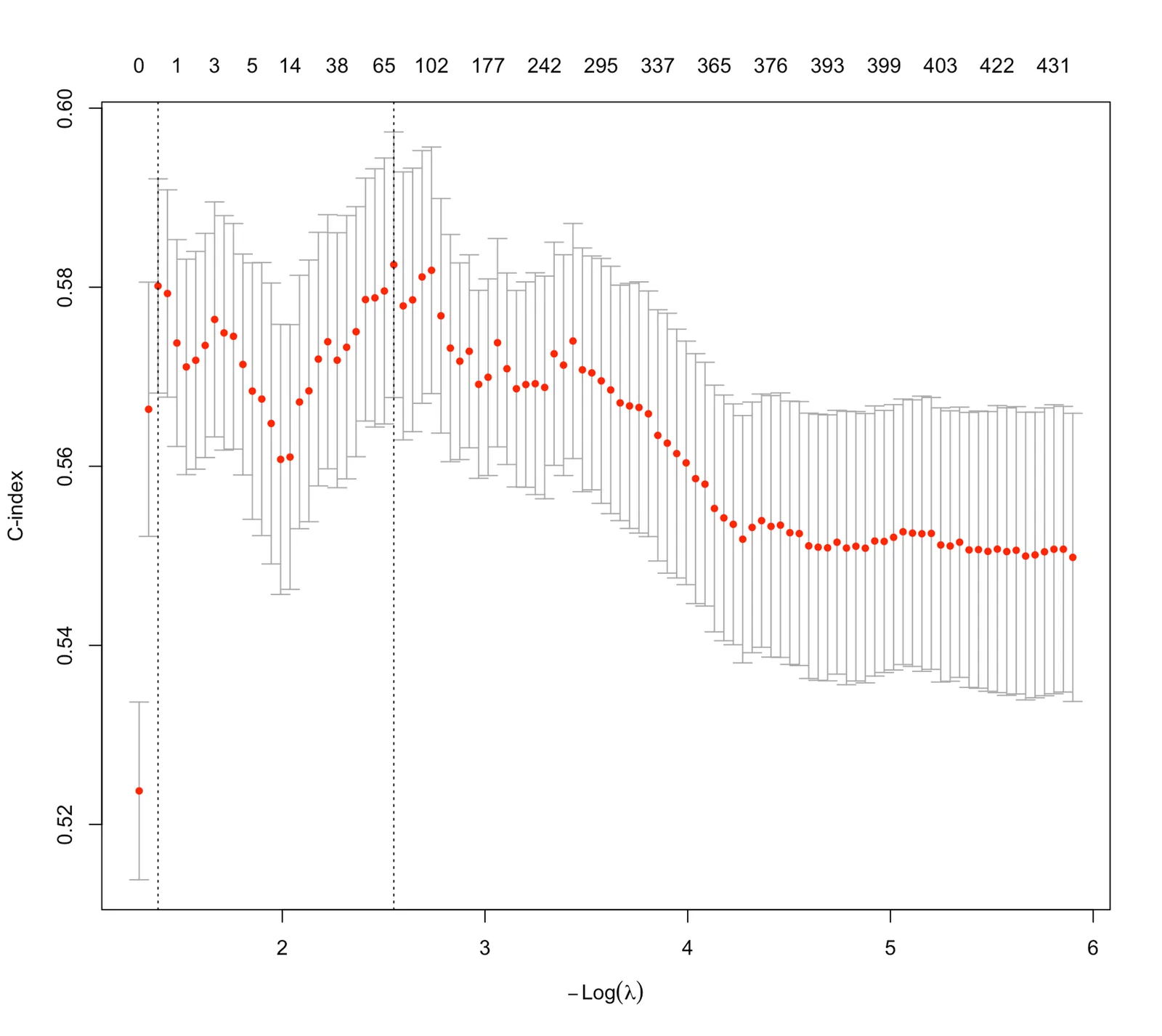
Ten-fold cross-validation results demonstrating concordance index (Harrell C-index) across lambda values Dashed vertical lines indicate lambda.min (right) and lambda.1se (left). The lambda.min model achieved the highest cross-validated concordance index. Tick labels on the upper x-axis indicate the number of nonzero coefficients identified by the LASSO-penalized Cox regression for varying values of lambda. Matched survival and gene expression data from 414 GBM tumor samples in the training dataset were used in this analysis.

After preprocessing, 518 GBM patients and 12,042 transcriptomic features were retained for analysis. Cross-validation identified λ_min = 0.078 and λ_1se = 0.250 as the optimal regularization parameters. The maximum cross-validated concordance index was 0.583 for the λ_min model (Figure 1).

Coefficient path plots suggest progressive shrinkage of transcriptomic feature coefficients with increasing regularization strength, illustrating transcriptomic feature selection behavior within the LASSO framework (see Appendix 1). The relationship between regularization strength and model performance was further evaluated by examining the percentage of null deviance explained across lambda values (see Appendix 2). Lower levels of regularization were associated with higher deviance explained, reflecting greater model fit within the training data.

Using the λ_min model, 74 genes retained nonzero coefficients. Amongst these 74 genes, two of them, CLEC5A and RANBP17, were also previously reported in a separate analysis (performed by Yoon et al. (2023)) to be amongst a list of 33 genes that are significantly correlated with GBM patient prognosis [24]. The probability of this event occurring by random chance (i.e. if LASSO-penalized Cox regression were to assign nonzero coefficients to 74 genes at random) is calculated to be 3.726 × 10-5. More generally, the probability of finding at least two genes overlapping by random chance between the set of 74 genes identified by Cox regression and the set of 33 genes identified by Yoon et al. (2023) is calculated to be p = 0.0174 [24]. These findings suggest that the observed overlap between the identified transcriptomic features and previously reported candidate prognostic genes is unlikely to be attributable solely to random chance.

**Figure 2:**
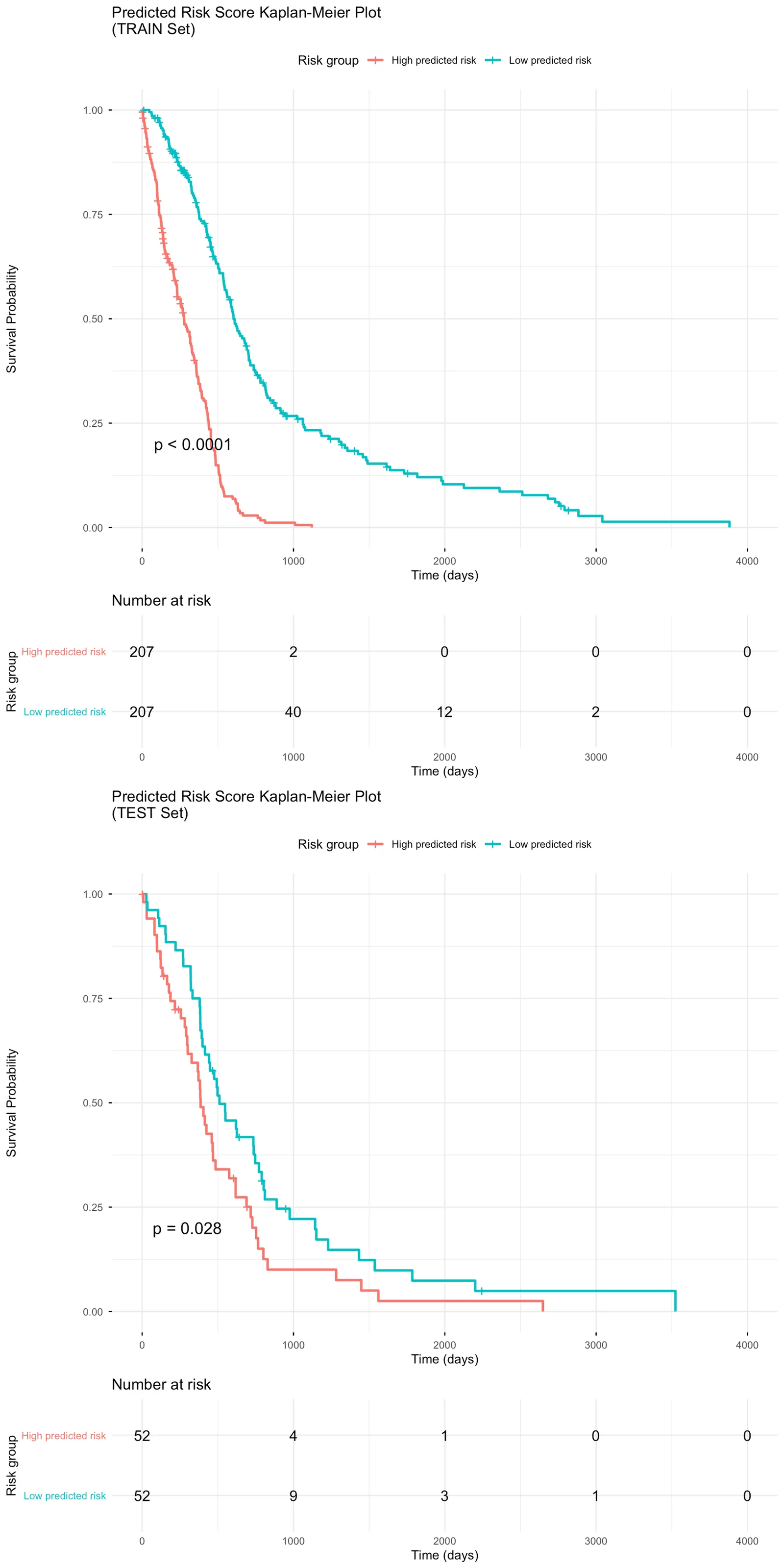
Kaplan-Meier survival analysis stratifying TCGA glioblastoma patients into high-risk and low-risk groups based on median-split LASSO-Cox predicted risk scores generated using the lambda.min model Results for the training dataset (n=414) are shown at the top, and results for the test dataset (n=104) are shown at the bottom. Patients in the high-risk group demonstrated significantly worse overall survival compared to patients in the low-risk group (log-rank p < 0.0001 in the train dataset, log-rank p < 0.05 in the test dataset). Risk tables indicate the number of patients remaining at risk over time. TCGA: The Cancer Genome Atlas

The predictive scores produced by the λ_min model demonstrated moderate but reproducible risk stratification performance in stratifying GBM patient survival. Four hundred and fourteen patients from the training dataset were stratified into low- vs high-risk groups based upon the median predicted risk score, and patients in the high-risk group exhibited significantly worse overall survival outcomes compared to patients in the low-risk group (p < 0.0001) (Figure 2). A similar outcome was observed in the test dataset (n=104), albeit with a lesser magnitude of separation (p < 0.05) (Figure 2).

**Figure 3:**
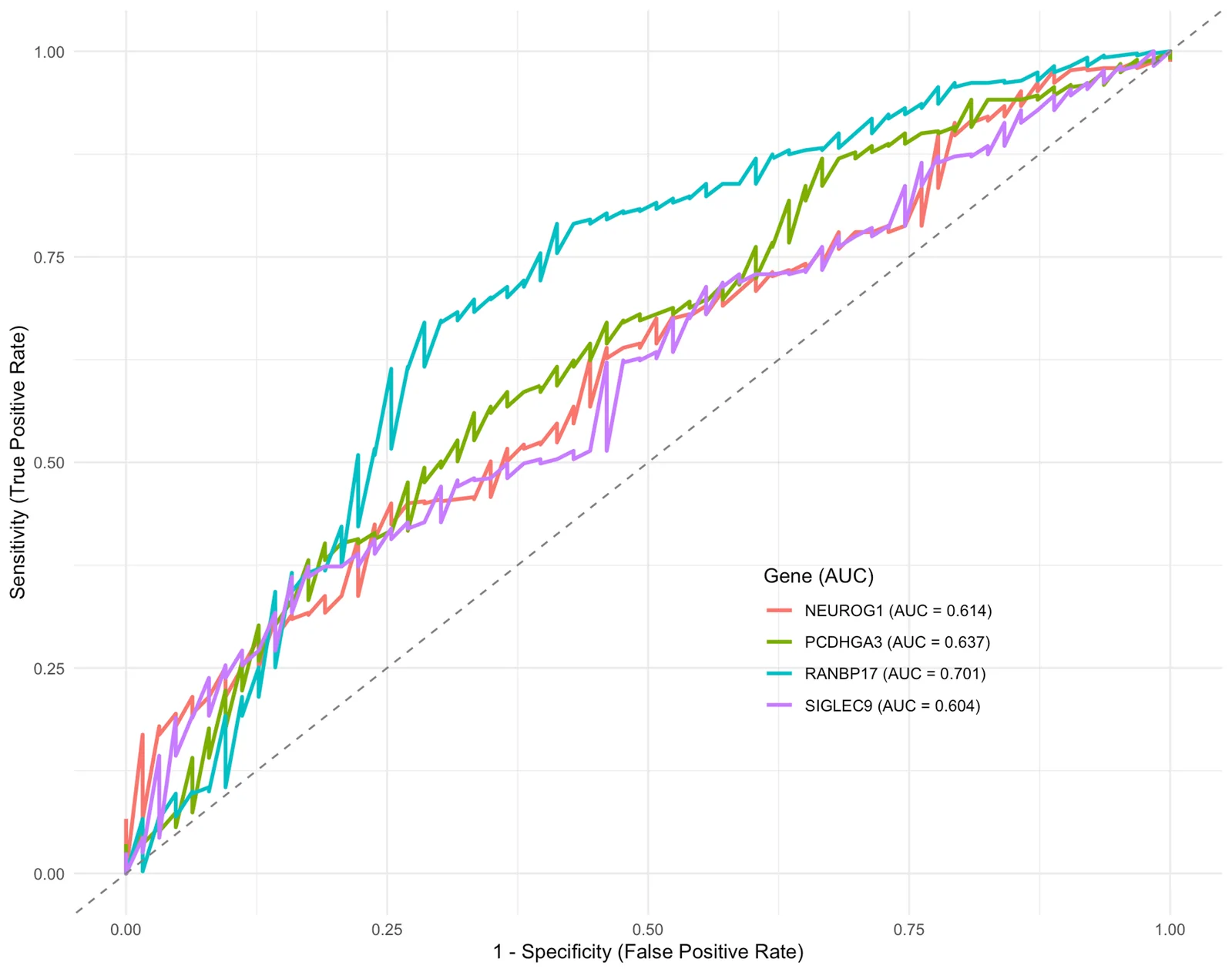
Receiver operating characteristic (ROC) curves for NEUROG1, PCDHGA3, RANBP17, and SIGLEC9 in the classification of long-term survival status AUC values ranged from a low of 0.604 for SIGLEC9 to a high of 0.701 for RANBP17. Survival data from 454 GBM patients (whose overall survival time was not right-censored prior to the cutoff of 2.5 years) were used in this analysis. AUC: Area under the curve; GBM: glioblastoma

Four candidate genes (PCDHGA3, RANBP17, SIGLEC9, and NEUROG1) with large coefficient magnitudes were selected for exploratory post hoc evaluation of their association with long-term survival. Exploratory ROC analyses were then performed for each of these genes by training a logistic regression classifier to predict patient survival past 2.5 years based upon expression levels of these four genes (Figure 3). AUC values ranged from a low of 0.604 for SIGLEC9 to a high of 0.701 for RANBP17. This exploratory AUC performance was similar in magnitude to that achieved in the previously mentioned analysis performed by Yoon et al. (2023) [24], where the AUC achieved by their top candidate gene predictors (also for the task of predicting survival past 2.5 years) ranged from a low of 0.605 to a high of 0.711.

## Discussion

In this study, we applied LASSO-regularized Cox proportional hazards regression to TCGA GBM transcriptomic data to identify a sparse transcriptomic signature associated with overall survival. The resulting model demonstrated separation between higher-risk and lower-risk patient groups and identified multiple transcriptomic features associated with survival outcomes. These findings support the feasibility of applying penalized transcriptomic survival modeling approaches for prognostic stratification in GBM.

GBM remains one of the most aggressive primary central nervous system malignancies, characterized by substantial molecular heterogeneity and poor overall prognosis despite multimodal therapy including surgical resection, radiation therapy, and temozolomide chemotherapy [3,25]. Increasing interest has therefore focused on identifying molecular biomarkers capable of improving prognostic stratification and informing precision oncology strategies in GBM [26].

Prior studies have similarly demonstrated the prognostic relevance of transcriptomic biomarkers in GBM. Yoon et al. (2023) [24] identified 33 genes associated with improved long-term survival in GBM using transcriptomic analyses. While only partial overlap was observed between previously reported genes and those retained in the present model, these differences likely reflect variation in preprocessing methods, cohort composition, statistical methodology, feature selection thresholds, and regularization approaches. Nevertheless, both studies support the broader concept that transcriptomic signatures contain potentially meaningful prognostic information in GBM.

Our findings are also consistent with other transcriptomic prognostic modeling studies in GBM that have identified sparse gene signatures associated with patient survival using penalized regression and machine learning approaches. Although the specific genes selected vary substantially across studies because of differences in transcriptomic platforms, feature-selection strategies, preprocessing methods, and patient cohorts, these investigations collectively support the concept that transcriptomic data contain meaningful prognostic information in GBM. The variability between reported signatures further highlights the need for external validation before candidate biomarkers can be considered clinically applicable [12,27].

LASSO regularization is particularly advantageous in transcriptomic survival modeling because it enables simultaneous variable selection and coefficient shrinkage in high-dimensional datasets where the number of transcriptomic variables greatly exceeds the number of patients [28]. Compared with traditional multivariable Cox regression approaches, penalized regression methods reduce overfitting risk while improving interpretability and model stability. This sparse modeling framework may therefore be well suited for transcriptomic biomarker discovery in cancer datasets with large feature spaces and relatively limited sample sizes.

Interestingly, several genes demonstrating strong univariate discriminatory performance in ROC analyses were not retained among the highest-weighted LASSO coefficients. This likely reflects the distinction between univariate predictive performance and multivariable penalized survival modeling. While ROC analyses evaluate genes independently, LASSO Cox regression identifies features contributing unique prognostic information after accounting for correlated transcriptomic patterns across the broader model. Consequently, genes with strong isolated discriminatory performance may provide overlapping or redundant information in multivariable transcriptomic models.

Several genes retained within the final model have also been implicated in GBM biology in prior studies. For example, OSMR has previously been associated with mesenchymal signaling pathways and aggressive GBM phenotypes [29]. Similarly, CLPTM1L has been implicated in tumor proliferation and therapeutic resistance across multiple malignancies [30]. These findings provide biological context supporting the potential relevance of the identified transcriptomic signature.

Although preliminary, these findings suggest that sparse transcriptomic survival models may contribute to future precision oncology frameworks for GBM. Integration of transcriptomic risk signatures with clinical, radiographic, and molecular variables may improve individualized prognostication and may ultimately contribute to treatment stratification following independent validation in future studies [27]. Future studies incorporating external validation cohorts and multimodal molecular and clinical data may further improve prognostic performance and clinical applicability.

This study has several limitations. First, analyses were performed retrospectively using publicly available TCGA data, which may limit generalizability to broader clinical populations. External validation using independent transcriptomic cohorts was not performed. Transcriptomic preprocessing methods and potential batch effects may influence feature selection and coefficient stability. Additionally, the relatively limited sample size compared with the dimensionality of transcriptomic features introduces potential risk of overfitting despite regularization. Formal assessment of the proportional hazards assumption was not performed and should be evaluated during future external validation studies. Furthermore, because transcriptomic measurements were generated using the TCGA HG-U133A microarray platform rather than RNA sequencing, low-abundance transcripts and certain transcript isoforms may not have been fully captured. Finally, biological and experimental validation of the identified genes was beyond the scope of the present study.

## Conclusions

LASSO-regularized Cox regression identified a sparse transcriptomic signature associated with OS in GBM and demonstrated the feasibility of penalized regression for feature selection in high-dimensional transcriptomic data. The resulting model demonstrated reproducible separation between higher- and lower-risk groups within an internal held-out test cohort between high-risk and low-risk patient groups and identified several candidate transcriptomic features warranting further investigation. These findings support the broader application of penalized survival modeling approaches for high-dimensional transcriptomic datasets, where simultaneous feature selection and risk prediction are essential. While additional validation is required before clinical implementation, the present study suggests the feasibility of leveraging transcriptomic data to generate interpretable prognostic models in GBM. Continued integration of molecular profiling and survival modeling may contribute to future efforts aimed at improving patient stratification and future precision oncology strategies for this challenging disease.

## Figures and Tables

**Figure 4:**
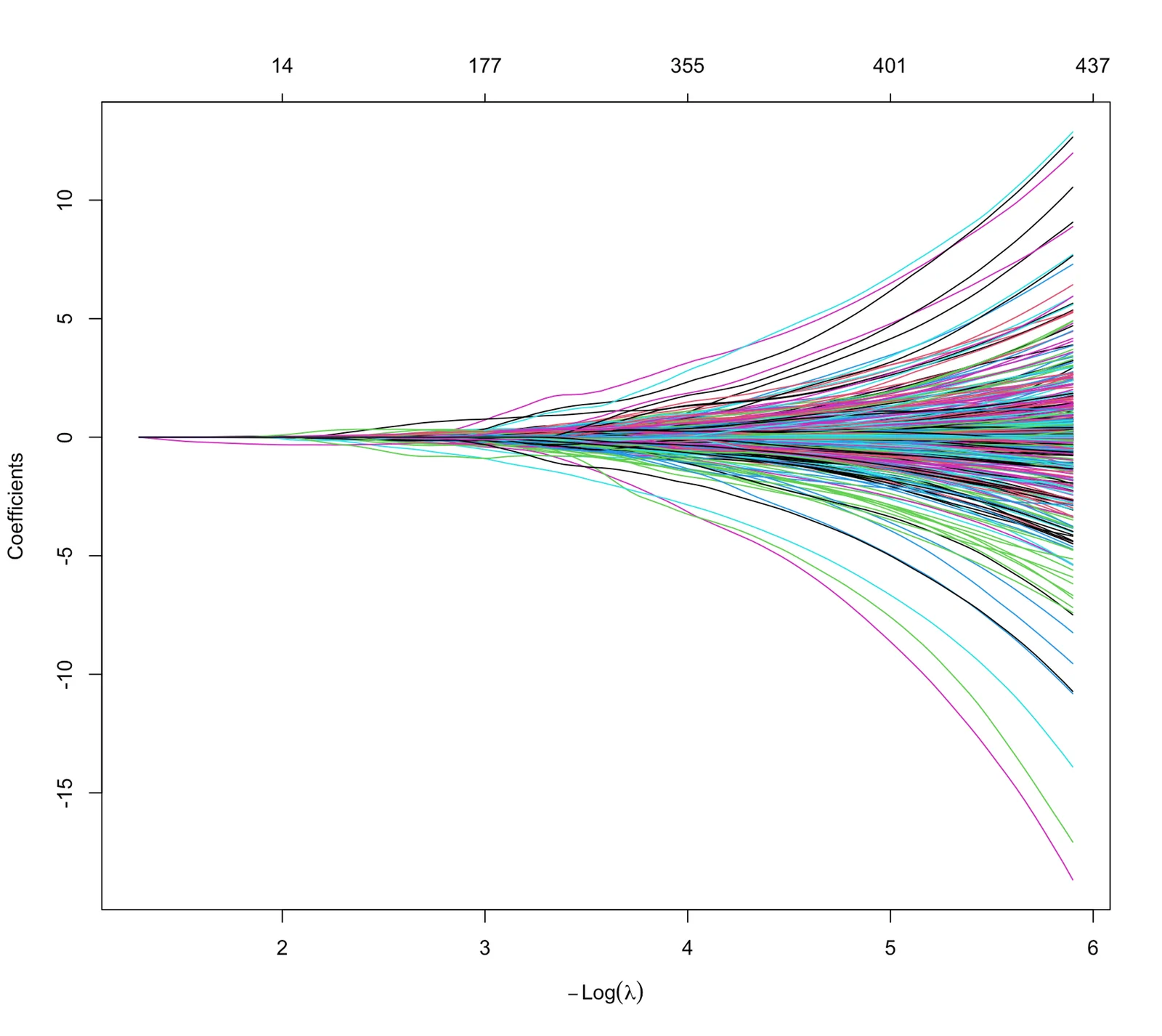
Coefficient trajectories for transcriptomic features across varying levels of regularization (log(λ)) demonstrating progressive shrinkage of coefficients toward zero as regularization strength increased Axis ticks on the upper x-axis indicate the quantity of nonzero coefficients and axis ticks on the y-axis indicate values of each coefficient.

**Figure 5:**
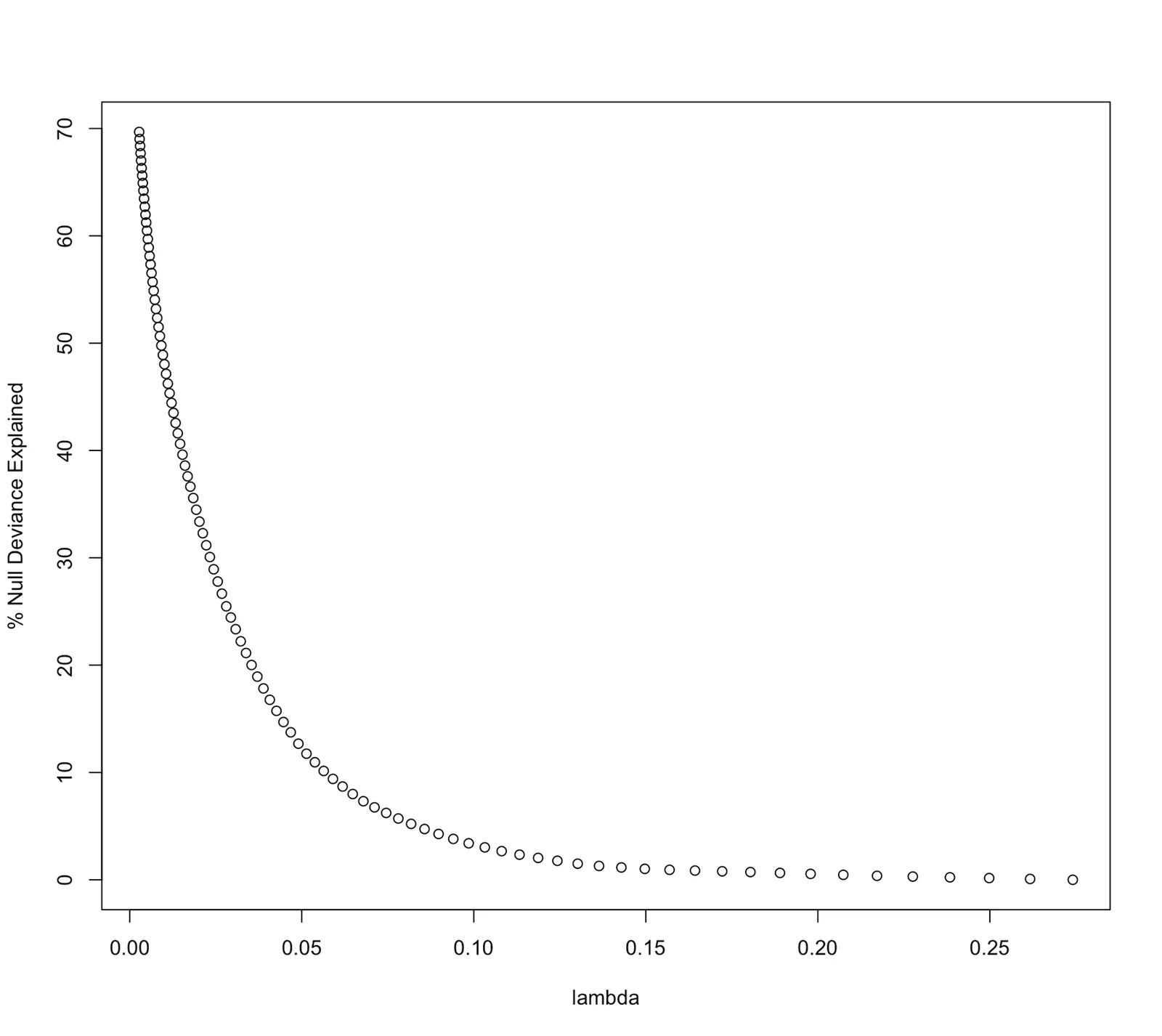
Relationship between lambda and percentage of null deviance explained by the LASSO-regularized Cox regression model Lower levels of regularization were associated with increased deviance explained and improved overall model fit. LASSO: Least Absolute Shrinkage and Selection Operator

## Appendices

Methods

LASSO-regularized Cox proportional hazards regression was performed using the glmnet package in R. The regression model was tasked with solving the following optimization problem:

$\min_{\beta_0,\beta} \frac{1}{N}\sum_{i=1}^{N} l(y_i,\beta_0+\beta^T x_i)+\lambda \lVert \beta \rVert_1$

where $l(y_i,\eta_i)$ is the negative log-likelihood contribution for observation *i* and *λ* controls the regularization strength. Furthermore, the likelihood function for the Cox proportional hazards model is specified as follows. Input data is provided in the form $(y_1,x_1,\delta_1),\ldots,(y_n,x_n,\delta_n)$ where $y_i$ represents the observed time and $\delta_i$ equals one if death is observed and equals zero otherwise (right-censored time). We also let $t_1&lt;t_2&lt;\ldots&lt;t_m$ be the increasing list of unique failure times, and let *j(i)* denote the index of the observation failing at time $t_i$.

The Cox model assumes the hazard function

$h_i(t)=h_0(t)e^{x_i^T\beta}$

where $h_i(t)$ is the hazard for patient *i* at time *t*, $h_0(t)$ is a shared baseline hazard, and *β* is a fixed-length vector of coefficients. The likelihood function is thus defined by

$L(\beta)=\prod_{i=1}^{m}\frac{e^{x_{j(i)}^T\beta}}{\sum_{j\in R_i}e^{x_j^T\beta}}$

where $R_i$ is the set of indices *j* with $y_j\geq t_i$ (those at risk at time $t_i$). Implementation of the penalized Cox regression framework followed the glmnet methodology described previously [2-4].

Appendix 1

Appendix 2
